# Depression, Quality of Life and Medication Use Among Patients With Chronic Pain: A Cross-Sectional Study

**DOI:** 10.1155/prm/6610938

**Published:** 2025-08-22

**Authors:** Parbati Thapa, Raj Kumar Thapa, Bhuvan K. C., Sudesh Gyawali, Shaun Wen Huey Lee

**Affiliations:** ^1^Pharmaceutical Sciences Program, School of Health and Allied Sciences, Pokhara University, Kaski, Pokhara, Nepal; ^2^School of Pharmacy, Monash University Malaysia, Subang Jaya, Selangor, Malaysia; ^3^Pharmacy Program, Gandaki University, Kaski, Pokhara, Nepal; ^4^School of Clinical Sciences, Queensland University of Technology, Brisbane, Australia; ^5^College of Public Health, Medical, and Veterinary Sciences, James Cook University, Townsville, Queensland, Australia; ^6^Department of Pharmacology, Manipal College of Medical Sciences, Pokhara, Nepal; ^7^School of Pharmacy, Faculty of Health and Medical Sciences, Taylor's University, Subang Jaya, Selangor, Malaysia; ^8^Asian Centre for Evidence Synthesis in Population, Implementation and Clinical Outcomes (PICO), Health and Well Being Cluster, Monash University Malaysia, Bandar Sunway, Subang Jaya, Selangor, Malaysia; ^9^Global Asia in the 21st Century (GA21) Platform, Monash University Malaysia, Bandar Sunway, Subang Jaya, Selangor, Malaysia

**Keywords:** chronic pain, depression, Nepal, quality of life

## Abstract

**Background:** Chronic pain is one of the most common reasons for seeking medical care and is associated with depression and reduced quality of life. This study aims to explore the association of depression and quality of life with chronic pain, and medication management among patients with chronic pain.

**Methods:** A cross-sectional study was conducted among patients visiting the outpatient departments of two tertiary care hospitals in Pokhara, Nepal. Validated questionnaires were used to collect information regarding socio-demographics, pain intensity (face pain scale), quality of life (EQ-5D-3L), depression (PROMIS depression questionnaire) and medications prescribed for pain management. Descriptive statistics, univariate analysis, correlation and multiple regression were used to analyse the data.

**Results:** Three hundred eighty-five participants were enrolled in the study, and most were females 248 (64.4%). Low back pain (*n* = 96; 24.9%) was the most commonly reported pain condition. The participants' mean pain and quality of life scores were 4.5 ± 1.97 and 0.59 ± 0.37, respectively. NSAIDs were the most prescribed medication. About 25.2% of the participants had mild, 25.5% moderate and 3.4% severe depression. A significant difference in depression and quality of life score was observed between genders (*p* < 0.001), among participants with different education levels (*p* < 0.001), with comorbidity (*p* < 0.001) and pain duration (*p* < 0.001). A significant reverse association of quality of life with depression (*β* = −0.326, *p* < 0.001), pain score (*β* = −0.292, *p* < 0.001) and duration of pain (*β* = −0.208, *p* < 0.001) was observed.

**Conclusion:** The quality of life among patients with chronic pain was correlated with the pain score, depression score and duration of pain.

## 1. Introduction

Pain is an unpleasant sensory and emotional experience associated with or resembling that associated with actual or potential tissue damage [1]. Pain, particularly chronic pain, is one of the most common reasons for seeking medical attention and a significant contributor to disability [2]. Osteoarthritic pain, back pain and headache are three of the top 10 reasons for seeking care for pain [3]. Chronic pain prevalence rates differ across countries, with a pooled chronic prevalence estimate of 43.5% in the United Kingdom and a lower prevalence of 20.4% in the United States. Low-middle-income countries have a prevalence range of 33.9%–41.1%, while Nepal has a higher prevalence of 48%–50% [4].

Chronic pain leads to various complications, usually physical dysfunction and altered mental states, with the progression of the pain [5]. Depressive symptoms are frequently reported among chronic pain patients, which potentiate pain, cause sleep deprivation and affect daily activities [6]. These affect family, social and workplace relationships and reduce quality of life [7]. Therefore, timely identification and management of such complications is crucial in handling chronic pain conditions.

As pain is a dynamic consequence of psychological, biological and social factors, guidelines have recommended an interdisciplinary treatment using a personalised approach [8]. The US Veteran Health Administration advocates that care should begin with the least intensive service and slowly progress towards more specialised care via patient-centered care [9]. However, many chronic pain cases are inadequately managed, adversely affecting patients' physical and emotional well-being, work efficiency and quality of life [10].

Pain management, as a discipline, is still evolving in Nepal. Patients with pain are either treated in the outpatient department or the emergency department (ED) of the hospitals. Likewise, there is a lack of appreciation when it comes to the impact of chronic pain on the quality of life and mental health of patients. There is scant literature on chronic pain, its types and quality of life, and the patient's mental health. Against this backdrop, this study aims to determine the type of chronic pain patients visiting the hospitals in Nepal and the associated factors such as depression, quality of life and the medication used for the management.

## 2. Methods

This study follows the Strengthening the Reporting of Observational Studies in Epidemiology (STROBE) recommendations for reporting observational studies [11].

### 2.1. Study Design and Study Site

This cross-sectional study was conducted in outpatient departments of two tertiary care hospitals in Pokhara, Nepal, from June 2021 to November 2021. These hospitals are well equipped to provide affordable healthcare services to the public. They have a high patient flow and are accessible to patients from rural and urban areas.

### 2.2. Study Population

The study population included patients with chronic pain complaints visiting outpatient departments of the hospitals.

### 2.3. Inclusion Criteria

Patients with complaints of chronic pain (pain persists for 3 months and more), aged 18 years and above, and willing to participate in the study were included.

### 2.4. Exclusion Criteria

Patients with cancer pain, cognitive impairment and those who could not understand the questionnaire were excluded.

### 2.5. Sample Size

The sample size was calculated with the Rao soft calculator using the previously reported prevalence of chronic pain in Nepal, 50% [12], with a 5% margin of error and a 95% confidence interval. The minimum required sample size for this study was estimated to be 385.

The high-quality prevalence data on chronic pain is limited, particularly for LMICS, due to the lack of standardised assessment tools and the complex interplay of social and psychological factors. The study by Walters et al. was selected due to their methodological rigour and relevance to LMICs [12]. The study reported the findings from the Vanderbilt Global Pain Survey (VGPS) in India and Nepal, a validated and standardised tool, enhancing the credibility of their findings. Calculating the sample size on the estimate, we aimed to improve the accuracy and generalisability of our sample size calculation.

### 2.6. Variables

The primary variables of interest in this study were the sociodemographic details including age, gender, occupation, associated comorbidities, and medications used. Likewise, continuous outcomes measured were the pain, depression and quality of life scores. Age, gender, and occupation have an association with chronic pain, and variations on these issues will affect the pain perception, intensity of pain, reporting, and coping mechanism. Similarly, associated comorbidities lead to increased pain intensity, reduced quality of life, and poorer treatment outcomes, and chronic pain for a longer duration leads to depression. Hence, it is crucial to assess these variables in our study.

### 2.7. Data Collection Tool and Scoring System

Information on chronic pain and its management was assessed using a validated questionnaire to identify the sociodemographic information, pain characteristics (duration of pain and pain site) and medications used. This was supplemented with questionnaires to assess pain score, depression and quality of life, as described below.

#### 2.7.1. Face Pain Scale

The face pain scale, translated to Nepali language, was used to measure the pain score. It consisted of a facial pictorial representation, with each face showing more pain from left to right. Each face was represented by a score of 0, 2, 4, 6, 8 and 10, where ‘0' means ‘no pain' and ‘10' represents ‘very much pain' [13]. The participants were clearly explained on what each face and number represents on the face pain scale. Participants were requested to point out the face that shows how bad their pain is at present and the scores were recorded accordingly.

#### 2.7.2. Patient-Reported Outcomes Measurement Information System (PROMIS) Depression Questionnaire

Depression was assessed using PROMIS depression 8b short-form questionnaire. A Nepalese-translated version of the questionnaire was used. The reliability and validity of the questionnaire have been described previously [14]. The translation of the PROMIS depression questionnaire in the Nepalese language was performed using the Functional Assessment of Chronic Illness Therapy (FACIT) translation methodology, which includes forward and backward translation, independent reviews, harmonisation, cognitive debriefing and proofreading. Debriefing was performed among six adults with chronic musculoskeletal pain. The translated questionnaire was relevant and comprehensive, which could be used for research and clinical settings [15].

The tool assesses the self-reported negative mood (sadness, guilt), views of self (self-criticism, worthlessness), social cognition (loneliness, interpersonal alienation) and decreased positive affect (loss of interest, meaning and purpose). It has eight items with five responses, options ranging in value from one to five on the Likert scale. A depression score of below 55 was considered ‘within normal limit', 55 to 59.9 as ‘mild', 60 to 69.9 as ‘moderate' and 70 and above as ‘severe' [16].

#### 2.7.3. Quality of Life

The Nepalese version of the EQ-5D-3L (EUROQOL) measure was used for the health-related quality of life [17, 18]. It assesses the quality of life based on five dimensions: mobility, self-care, usual activities, pain/discomfort and anxiety/depression. Each dimension has three levels: no problem ‘1', some problem ‘2' and extreme ‘3'. A single index value was calculated in the ‘EQ-5D-3L Crosswalk Index Value Calculator' using weights of the United Kingdom as the reference from the five dimensions of EQ-5D [19, 20]. The EQ-5D index ranges from 0 to 1, where 0 represents severely ill, and 1 indicates perfect health. No problems on all five dimensions (11111) represent perfect health with the value assigned as 1, and severe health problems in all dimensions (33333) represent very severe health states with the value assigned as near to 0. A higher EQ-5D index score indicates a better health-related quality of life.

### 2.8. Validity and Reliability of the Tools

Nepalese versions of the face pain scale, PROMIS and EUROQOL EQ-5D-3L have shown excellent reliability and validity for assessing health outcomes, including the pain domain in Nepalese individuals. The tools have been systematically translated, adapted and evaluated for their measurement properties, which ensures their reliability and validity for their use with confidence in research and clinical settings.

### 2.9. Process of Data Collection and Data Management

A pilot study was conducted among 30 participants to ensure the feasibility and appropriateness of the chosen tools. Although the questionnaires were deemed acceptable, poor literacy among participants was challenging. To ensure uniformity, it was decided that a data collector would administer all questionnaires.

During the pilot study, the response rate for the questions was high, almost 90%, suggesting strong initial engagement and feasibility. Participants could complete or respond the questionnaire in an average of 15 min and were fine with the time, indicating the acceptability of the time required to complete the questionnaire. Participants were asked to rate the questions for clarity, relevance, understandability and acceptability on a Likert scale (1 = strongly disagree to 5 = strongly agree). Majority of the participants responded to agree or strongly agree, suggesting that the questionnaire was clear, relevant, understandable and acceptable.

The principal investigator, senior clinical pharmacist and clinicians provided training to the data collectors to ensure consistency in collecting the information and reduce the bias. They were trained to act empathetically, professionally and sensibly while handling the questions related to pain, mental health and quality of life.

### 2.10. Participant Recruitment

Patients visiting the outpatient department with the complaint of pain were screened based on the inclusion and exclusion criteria. Selected patients were then informed about the purpose and study objectives. Written informed consent was obtained from literate patients, and the thumbprint technique was used for illiterate participants.

### 2.11. Statistical Analysis

Descriptive statistics were calculated for all variables. Univariate comparisons of quality of life (EQ-5D-3L) and PROMIS depression scores were performed using the Mann–Whitney *U* and Kruskal–Wallis tests. Spearman's correlation coefficients (*r*) were used to test the association of two continuous measures: age, pain score, depression score and quality of life score. Linear regression analysis determined the independent factor associated with the quality of life score (EQ-5D-3L). Standardised regression coefficients were measured to determine the effect of independent variables, and *R*-squared was reported as the percent of the variance explained by the model. The dependent variable, quality of life score, was compared with the independent variables, pain duration, pain score and depression score, in a regression model. The variables were selected on their clinical relevance among the pain population and association with quality of life score based on the prior findings. Covariates (e.g., age, gender) were not reported as part of this model, which limits the ability to account for potential confounding factors. Linearity and multicollinearity were assessed before conducting the regression analysis. The results supported the assumption of linearity, and no multicollinearity was detected, as all variance inflation factor (VIF) values were within acceptable limits.

All data were analysed using the Statistical Package for Social Science (SPSS, Version 26.0) software. A *p* value less than 0.05 was considered to be statistically significant.

### 2.12. Ethical Consideration

Ethical approval for the study was obtained from the Nepal Health Research Council (Registration number 211/2020). Permission to collect the data was obtained from institutional review committees of the respective hospitals. All patients provided written informed consent. Prior to data collection, all participants were thoroughly informed about the study's objectives and their right to withdraw from participation at any point without any repercussions. They were also assured that all information provided would be treated with strict confidentiality. To ensure anonymity, participants' names were not recorded on the questionnaires. The collected data were securely stored and used exclusively for research purposes. Coding was performed during the data entry, and the entered data were encrypted with passwords for safety and access control.

This study adhered to the ethical guidelines outlined in the Declaration of Helsinki.

## 3. Results

### 3.1. Descriptive Data

A total of 400 participants were recruited for the study; after excluding 15 participants with missing data, 385 were included in the final analysis (Table 1). Out of 15 participants excluded from the study, almost 9 participants initially consented to participate; however, they could not complete the survey as they needed to leave early to travel back to their homes. Participants enrolled in the study were from neighbouring districts like Syangja, Parbat and Tanahu, where they needed to travel for 1–3 h back home. For the remaining 6 participants, incomplete information was primarily observed in depression and quality of life assessment, where they opted not to answer.

Most participants were females (*n* = 248; 64.4%), with a median age of 49 years (range 18–91 years). The most common primary reason for seeking medical care was musculoskeletal pain, particularly back and knee pain. Abdominal pain and headache were among other commonly reported reasons. The mean pain score of the participants was 4.5 ± 1.97, with half of them (*n* = 216; 56.1%) having pain complaints for at least 3–11 months. Participants were on a mean of 3.8 medications for pain, with nonsteroidal anti-inflammatory drugs (NSAIDs) being the most prescribed medication. Likewise, steroids (11.4%), anticonvulsants (11.3%) and muscle relaxants (21.3%) were prescribed to some extent for chronic pain management; however, the use of opioids was significantly less (0.5%) (Table 2).

### 3.2. Depression Among Participants With Chronic Pain

About 50% of the participants had mild (25.2%) to moderate depression (25.5%). The mean ± SD depression score among participants was 55.28 ± 9.22. According to the results of the univariate analysis, there was a significant positive correlation between depression and age (*r* = 0.19, *p* < 0.001), as well as pain score (*r* = 0.314, *p* < 0.001). Female participants with no formal education, longer pain duration and comorbidity reported higher depression scores (*p* < 0.001), as shown in Table 3.

### 3.3. Quality of Life Among Participants With Chronic Pain

Assessment of different components of quality of life revealed that the most common complaints by participants were related to their pain/discomfort (*n* = 246; 63.9%), anxiety/depression (*n* = 142; 36.9%) and mobility (*n* = 105; 27.3%). Other complaints include issues with self-care (*n* = 68; 17.7%) and usual activities (*n* = 127; 33%) (Table 4). We noted some differences in the self-reported quality of life, with poorer reported quality of life scores among females than males (0.73 vs. 0.76; *p* < 0.001). Likewise, participants with no formal education presented with comorbidity, longer pain duration, higher pain score and depression score and had a low self-reported quality of life (*p* < 0.001). Multiple regression analysis showed that pain duration (*β* = −0.208, *p* < 0.001), pain score (*β* = −0.292, *p* < 0.001) and depression score (*β* = −0.326, *p* < 0.001) were independently associated with the quality of life score (Table 5).

## 4. Discussion

The current study assessed patient characteristics, the association of depression and quality of life with chronic pain and medication management among those visiting tertiary care hospitals of Nepal. Several studies have reported patient characteristics and pain severity among chronic pain patients [4, 12, 21]. However, to the best of our knowledge, this is the first study to explore the association of quality of life with depression and chronic pain in Nepal.

Three hundred and eighty-five participants were enrolled in this study. The chief complaint from the participant seeking care was musculoskeletal pain, followed by abdominal pain and headache, especially among females. Low back pain (*n* = 96, 24.9%) and knee pain (*n* = 85, 22.1%) were the commonly reported pain. Nearly 50% of the population had the complaint of either knee pain or low back pain, which is clinically significant. This might be due to several factors, including age, gender, physical activity, lifestyle, and occupation among Nepalese participants [22].

This finding is similar to the one reported by Bhattarai and colleagues in the community-based study in Nepal, where most participants had musculoskeletal-related chronic pain and higher reporting among females [21]. Likewise, Dasgupta and colleagues reported a higher prevalence of musculoskeletal pain (knee pain) and preponderance among female patients visiting primary healthcare clinics in Malaysia [23]. Both of the refereed studies share the common study design, i.e., the cross-sectional study. Likewise, the study by Dasgupta has been conducted in the same settings and healthcare facilities, which might have contributed to the similar findings.

Low back pain is a global problem; its prevalence was estimated at 7.5% of the worldwide population in 2017 [24]. Low back and knee pain are common chronic pain problems in Nepal and are highly prevalent [21, 22, 25]. However, the higher prevalence of chronic pain among females is unclear. Laboratory research has revealed that women are more sensitive to experimental pain stimuli than men [26]. In their review study, Fillingim and colleagues reported that low intensity of different pain stimuli, pressure, heat, cold, electric and ischaemic, could easily provoke pain among females compared to males [27]. The exact mechanism to explain the gender difference is difficult; however, it is suggested that multiple factors, including endocrinological factors, cognitive and affective states, body size and functional capacity, and occupational status, might have some roles [28]. Hormones, especially sex hormones, affect pain regulation. The fluctuation of hormones, especially estrogen and progesterone, across the menstrual cycle, pregnancy and menopause significantly exacerbates chronic pain experiences [29]. Clinical and preclinical studies have suggested the protective effects of testosterone against pain among males; however, mixed effects were observed with female hormones, estrogen [30, 31].

It could also be that females are more inclined to report health problems, are involved in performing repetitive routine work, the prevalence of gender imbalance in domestic work and being frequent users of health care services might have contributed to the higher number of female participants with chronic pain [22].

The higher prevalence of musculoskeletal pain among the women warrants an effective pain management that includes biological, psychological and sociocultural aspects [32]. A well-designed, tailored mechanism for investigation, diagnosis, prevention and treatment with interdisciplinary and collaborative effort is even more effective. Delivering accurate and evidence-based information and promoting active participation in self-care is inevitable [33]. Tailored rehabilitation education and exercise could also significantly control the musculoskeletal pain [34].

The mean pain score of the participants was 4.5 ± 1.97, considered moderate. Shaygan and colleagues from Iran reported the mean pain score of 4.04 ± 2.49 and 4.26 ± 2.86 among adults diagnosed with chronic pain in their study's control and treatment groups, respectively, which is consistent with the findings of this study [35]. However, Nizar and colleagues in Malaysia reported pain intensity of 6.5 ± 1.40 among patients visiting the pain clinic with either cancer or noncancer pain [36]. While the exact reason for moderate pain scores among patients visiting the hospital is unclear, we postulate that this might be because patients with higher pain scores often visit the ED for its management. In a study by Baharuddin and colleagues, which examined the pain scores among ED patients, the authors found that most patients had a mean score of 6.8 [37]. Variation in reported pain scores between the studies is attributed to several factors including pain aetiology; either neuropathic or nociceptive, cancer pain or noncancer pain, pathophysiology, pain sites, differences in diseases stage and treatment approach. Variation is also dependent on the study population either inpatients or outpatients and on the type of pain assessment tools or scale [38]. In the above-referred studies, pain score was assessed using a numeric rating scale by Shaygan & Jaberi [35] and Baharuddin et al. [37]; however, Jalil et al. [36] used a visual analogue scale (VAS).

Most participants (*n* = 216, 56.1%) have a pain duration between 3 and 11 months, followed by 1–3 years. Majedi and colleagues reported that the average pain duration was 5 years among chronic pain patients [39]. Longer pain duration could be due to the delay in approaching treatment, the chronic nature of underlying conditions, delay in referral to the pain clinics and inadequate pain management.

Inadequate pain management could be a major concern for the longer pain duration among the participants in our study. It is further affected by several factors, including age, gender, socioeconomic status and perceived pain intensity by individuals. Limited or underprescription of analgesics due to comorbidities and fragile health conditions among the aged population could result in inadequate pain management and thus prolong the pain condition [39]. Gender, especially females, is reported to have longer pain duration, which could be due to the difference in pain sensitivity and analgesic response between genders. Especially in low- and middle-income countries, several barriers hinder effective pain management, including inadequate health care facilities, lower priority to pain management and inadequate education on pain management among health care professionals, which prolongs the pain duration [40].

Therefore, proper understanding and responding to the variability in pain duration is crucial for effective pain management. Especially in LMICs like Nepal, where chronic pain often remains underdiagnosed and undertreated, a supportive program for timely diagnosis, individualised care and long-term support is needed. An outreach program to the community focusing on knowledge about pain, myths and the importance of adherence to the treatment plan could be useful to promote timely reporting and management of pain [41].

NSAIDs were the most prescribed medication to manage pain in our study. Naproxen and aceclofenac were prescribed frequently for oral administration, and diclofenac gel was used for topical application. These findings are consistent with published reports from the United States [42], Switzerland [43], India [44] and Nepal [45]. However, Zin and colleagues from Malaysia reported opioids as the most prescribed analgesics in public hospitals, contrasting our findings [46]. The Centre for Disease Control (CDC) guidelines 2016 recommend caution when initiating opioids and avoiding their use as a first-line therapy [47]. The use of opioids in our study was very minimal. Opioids are internationally controlled medications for their potential abuse. Consumption of opioids in Asia is much less than in the United States and Europe [48]. Its consumption in Nepal is significantly less; physicians are reluctant to prescribe it due to fear of abuse and lack of training in pain management [49] attributing to the limited opioid prescriptions identified in our study. Higher consumption of opioids in Malaysia compared to our settings could be impacted by several factors; initially with the healthcare services, highly equipped healthcare facilities accessible for public and supported by health coverage, country-specific pain management guidelines and collaborative efforts between governmental, nongovernmental and other health organisation contribute to the availability and increasing trend of opioid use in Malaysia [50]. However, barriers to effective pain management and opioid use still exist in our settings, resulting in less opioid use. Hence, the findings suggest requirements of drug policy improvements to enhance the availability and accessibility of opioids in Nepal [51].

Antidepressants, anticonvulsants (pregabalin and gabapentin), steroids, and muscle relaxants were the other medications prescribed, and the prescription pattern aligns with the Scottish Intercollegiate Guidelines Network (SIGN) [52] guidelines 2019 for chronic pain management, which recommends using this medication for short- to medium-term treatment of chronic pain. Intra-articular steroids were used in a few patients with knee pain, and it has been shown to reduce pain and tenderness, especially in knee osteoarthritis [53]. Aroll and colleagues, in their meta-analysis study, reported the short-term improvement in knee osteoarthritis symptoms after intra-articular corticosteroid injection [54]. Muscle relaxants were combined with NSAIDs, and evidence suggests that they are effective for acute or chronic low back pain [55].

Our study confirmed the negative impact of higher pain scores and depression on patients' quality of life. A significant negative correlation was found between quality of life, pain score and depression. Patients with high pain scores and more depressive symptoms have a lower quality of life. Several studies support these findings, establishing the reciprocal relationship of chronic pain and depression with quality of life [56, 57]. Tsuji and colleagues reported depression and lower health-related quality of life scores among patients with chronic low back pain [58]. Elliott and colleagues reported an association of depression with health-related quality of life among chronic pain patients [59]. Likewise, Garnaes and colleagues, in a cross-sectional study among patients with musculoskeletal pain, reported the reduced health-related quality of life to be prevalent among females receiving a disability pension and several psychosocial factors [60].

Univariate analysis of depression among patients with chronic pain revealed a positive correlation with pain scores. A weak positive correlation value of 0.19 was observed between depression and age suggesting clinical nonsignificance; however, it was 0.314 between depression and chronic pain implying a moderate association and clinical significance. Chronic pain and depression are related and can co-occur [61, 62]. Depression among chronic pain patients could be explained based on several molecular mechanisms. The monoamine hypothesis states that the decreased availability of monoamine neurotransmitters such as serotonin and norepinephrine in the central nervous system may result in the occurrence of depression. Likewise, chronic pain deteriorates the dopamine activity in the midbrain. As a result, there are decreased dopamine levels and reduced expression of D2R, a dopamine receptor, which ultimately increases the chance of developing depression in relation to reduced monoamine neurotransmitters in the central nervous system [63]. Other possible factors could be that the pain gets worse with an increasing number of symptoms and severity, which will induce stress, and the chances of developing depression increase [64]. Increased inflammation due to increased levels of pro-inflammatory cytokines could also induce depression. In a chronic pain condition, stress could enhance the inflammatory response and increase the risk of depression [65].

About 50% of the participants in our study have mild to moderate levels of depression. Brooks and colleagues in England, and Muhammad and colleagues in India reported that participants with higher pain frequency and intensity have elevated depressive symptoms, consistent with our study's findings [66, 67]. Depression enhances the adverse effects on patients' outcomes, worsens functioning and reduces the response to treatment [68]. Bair and colleagues reported depression to be prevalent among 56% of patients with pain in orthopaedic clinics, while Zuraida and colleagues reported it to be 27% among patients with headaches [64, 69]. Likewise, Mallen et al. and Suija et al. reported 23%–35.5% cases of depression among patients with musculoskeletal pain [70, 71].

The discrepancies in reporting of depression among chronic pain patients across the studies could be due to the use of a variety of instruments and the techniques used for its assessment, either through clinical interview, psychiatric assessment, or the use of a questionnaire. Additionally, the differences are likely due to the types of pain conditions studied, research methodologies and characteristics of the study populations.

Zuraida and colleagues performed the study in an outpatient setting and used the structured clinical interview to assess depression, while Mallen and colleagues applied a cross-sectional survey, linked GP consultation and used a short questionnaire together with the Hospital Anxiety and Depression Scale among older adults with musculoskeletal pain [69, 70]. Likewise, Suija and colleagues assessed depression in primary medical care with the Composite International Diagnostic Interview approach [71].

Depression worsens the treatment outcomes among patients with chronic pain and vice versa, resulting in poor pain management and decreased treatment adherence. These factors lead to disability, an increase in healthcare utilisation and deteriorated mental health [64].

Therefore, a comprehensive approach addressing both these issues is necessary. A collective management approach using pharmacological therapy, cognitive behavioural therapy and mindfulness could be effective [72].

Depression and low self-reported quality of life were more prevalent among the elderly, females, participants with no formal education, patients with comorbidity and longer duration of pain. Females with chronic pain conditions are more prone to develop depression as contributed by social and biological factors [32, 73, 74]. Hormonal changes especially those occurring during menstruation, pregnancy and menopause are biological factors that can affect mood. On a social level, women are more likely to experience challenges such as poverty, difficulties balancing work and personal life, and gender-based violence, all of which can heighten the risk of depression [75]. Depression further affects the sleep pattern and induces stress [76]. Hence, coping with depression and related factors is quite challenging. A tailored management strategy considering pain, gender and mental health could be a beneficial.

In the study by Munce & Stewart [74], more depressed women rated their pain as ‘severe' than depressed men, suggesting gender differences in pain perception that might be associated with increased prevalence of chronic pain conditions among women resulting in the elevated rates of depression in women. In another study, the adults with higher levels of education reported less pain [77]. Educational attainment shapes access a wide range of financial and nonpecuniary resources that individuals can use to maximise health, reduce pain and thus prevent depression. Additionally, longer duration of pain causes trouble sleeping and stress, thus leading to depression caused by the common neuroplasticity changes [78]. The co-occurrence of chronic pain and depression among elderly patients is well established, given their high prevalence in this population. Specifically, research has shown that approximately 13% of elderly individuals suffer from chronic pain and depression [79]. Therefore, it is crucial to properly manage these co-morbid conditions using a combination of pharmacological and nonpharmacological approaches [80]. Depression among chronic pain patients is also often associated with severity, frequency, duration and number of symptoms [56]. In managing chronic pain, mental healthcare plays a critical role. Addressing comorbid conditions such as depression is crucial to achieving effective pain management outcomes. It can improve pain severity, overall functioning and pain perception and improve the quality of life [81]. Hadi and colleagues emphasised that poor pain management owing to failure to identify the multidimensional nature of chronic pain might be the reason for the poor quality of life among patients with chronic pain. Therefore, strategies to improve the quality of life and pain relief are crucial, especially in low- and middle-income countries where pain management is still challenging [82].

### 4.1. Strengths and Limitations

The present study's primary strength is the first of its kind study performed in Nepal to explore the association of quality of life with depression and chronic pain. Second, the reverse association of quality of life with depression, pain score and duration of life was determined that guides the necessity of intervention in appropriate pain management.

This study has some limitations. Firstly, the data were collected from only two study sites, which limits generalisability. Secondly, the findings are based solely on quantitative data, and including a qualitative approach would have provided a better understanding of depression and quality of life among chronic pain patients. A more rigorous study design that includes multiple approaches, sites and a large sample size is necessary to accurately assess pain and related domains and confirm the adequacy of treatment.

## 5. Conclusions

Musculoskeletal pain was the primary complaint, followed by headache and abdominal pain among the patients visiting the outpatient department of hospitals in Nepal. Females were found to have a higher prevalence of chronic pain, with most participants reporting moderate pain. The most frequently prescribed medication for managing chronic pain was NSAIDs. Participants with increased pain and depression scores reported a lower quality, which should be adequately addressed, especially in low- and middle-income countries. Hence, our study suggests that chronic pain can simultaneously influence both depression and quality of life, with depression further impacting quality of life.

## Figures and Tables

**Table 1:** Demographic details, pain scores, quality of life and depression scores of the study population.

Demographic details	Number	Percentage
Gender		
Male	137	35.6
Female	248	64.4
Age (median) (range)	49 (18–91)	
Age category		
18–25	31	8.1
26–35	36	9.4
36–45	89	23.1
46–55	89	23.1
56–64	72	18.7
65–74	44	11.4
75 and more	24	6.2
Education		
No formal education	152	39.5
Primary	47	12.2
Middle school/high school	144	37.4
University	42	10.9
Occupation		
Housewife	154	40.0
Teacher	13	3.4
Office employ	13	3.4
Farmer	79	20.5
Student	25	6.5
Retired	32	8.3
Business	37	9.6
Others	32	8.3
Smokers		
Yes	27	7.0
No	358	93.0
Alcohol		
Yes	45	11.7
No	340	88.3
Pain duration		
3–11 months	216	56.1
1–3 years	123	32
4–5 years	24	6.2
6 years and more	22	5.7
Pain sites		
Knee pain	85	22.1
Low back pain	96	24.9
Multiple sites pain	36	9.4
Shoulder pain	44	11.4
Leg/foot pain	23	6.0
Neck pain	13	3.4
Wrist/forearm/hand pain	15	3.9
Elbow pain	17	4.4
Hip pain	23	6.0
Abdomen	26	6.7
Headache	7	1.8
Comorbid condition		
No	263	68.3
Yes	122	31.7
Pain score mean ± SD	4.5 ± 1.97	
0 = no pain, 10 = very much pain		
2.00	75	19.5
4.00	196	50.9
6.00	71	18.4
8.00	27	7.0
10.00	16	4.2
Depression score mean ± SD	55.28 ± 9.22	
None to slight (less than 55)	177	46.0
Mild (55–59.9)	97	25.2
Moderate (60–69.9)	98	25.4
Severe (70 and over)	13	3.4
Quality of life (QoL) score (mean ± SD)	0.59 ± 0.37	
QoL visual analogue scale (0–100) mean ± SD	64.57 ± 22.93	

**Table 2:** Medications prescribed for management of pain.

Category	Medications	Number	Percentage
NSAIDS	Naproxen	136	35.3
	Aceclofenac	128	33.2
	Diclofenac gel	229	59.5
	Etoricoxib/celecoxib	46	11.9
	Piroxicam	1	0.2

Opioids	Tramadol	2	0.5

Analgesic and CNS stimulant/muscle relaxant	Paracetamol and caffeine	3	0.8
	Paracetamol and Chlorzoxazone	17	4.4

Anti-inflammatory	Diacerein	30	7.8

Steroids	Dexamethasone/methylprednisolone	44	11.4

Antidepressant	Duloxetine/amitriptyline	22	5.7

Anticonvulsants	Pregabalin	41	10.6
	Gabapentin	3	0.7

Muscle relaxants	Tizanidine	82	21.3

Vitamins and supplements	Calcium supplements	155	40.3
	Calcium and vitamin D	7	7
	Vitamin D	24	6.2
	Vitamin B12	22	5.7
	Multi-vitamin	18	4.7

Gastrointestinal agents	Sucralfate	17	4.4
	Antacids	4	1
	Hyoscine-N-butylbromide	10	2.6
	Rabeprazole	108	28
	Pantoprazole	222	57.7

Abbreviation: NSAIDs = nonsteroidal anti-inflammatory drugs.

**Table 3:** Univariate analysis results for EQ-5D and PROMIS depression scores.

	EQ5D score Median (IQR)	*p*-value	PROMIS depression score Median (IQR)	*p*-value
Gender				
Male	0.76 (0.16)	< 0.001^+^	54.3 (10.6)	< 0.001^+^
Female	0.72 (0.28)		57.1 (10.1)	
Age, *r*	−0.29^±^ 95% CI [−0.326, −0.131]	< 0.001^±^	0.19^±^ 95% CI [0.090, 0.297]	< 0.001^±^
Education				
No formal education	0.68 (0.50)	< 0.001^++^	57.9 (8)	< 0.001^++^
Primary	0.72 (0.81)		57.1 (9.3)	
Middle/high school	0.72 (0.14)		54.3 (9)	
University	0.79 (0.27)		51.2 (12)	
Smoking				
Yes	0.68 (1.34)	0.109^+^	57.1 (8.2)	0.136^+^
No	0.72 (1.34)		55.3 (11.8)	
Alcohol				
Yes	0.72 (0.40)	0.820^+^	55.3 (11.8)	0.334^+^
No	0.72 (0.21)		56.2 (11.8)	
Comorbid				
Yes	0.59 (0.17)	< 0.001^+^	57.1 (10.1)	< 0.001^+^
No	0.68 (0.75)		54.3 (9.9)	
Pain duration				
3–11 months	0.79 (0.10)	< 0.001^++^	54.3 (9)	< 0.001^++^
1–3 years	0.72 (0.28)		57.9 (11.3)	
4–5 years	0.60 (0.87)		61 (10.2)	
6 years and more	−0.05 (0.87)		62 (9.9)	
Pain scale, *r*	−0.374	< 0.001^±^	0.314	< 0.001^±^
Depression, *r*	−0.540	< 0.001^±^		

^±^Spearman's correlation.

^+^Mann–Whitney *U* test.

^++^Kruskal–Wallis test.

**Table 4:** Quality of life EQ-5D-3L.

	Mobility *N* (%)	Self-care *N* (%)	Usual activities *N* (%)	Pain/discomfort *N* (%)	Anxiety/depression pain/discomfort *N* (%)
No problem	279 (72.4)	310 (80.5)	248 (64.4)	67 (17.4)	171 (44.4)
Some problem	105 (27.3)	68 (17.7)	127 (33.0)	246 (63.9)	142 (36.9)
Extreme problem	1 (0.3)	7 (1.8)	10 (2.6)	72 (18.7)	72 (18.7)
Visual analogue scale	Mean ± SD 64.57 ± 22.93 Range (10–90)				

**Table 5:** Multiple linear regression analyses for EQ-5D.

	Regression coefficient	Standard error (SE)	Standardised regression coefficient (*β*)	*p*-value
Dependent variable: EQ-5D				
Pain duration	−0.094	0.019	−0.208	< 0.001
PROMIS depression score	−0.013	0.002	−0.326	< 0.001
Pain score	−0.056	0.008	−0.292	< 0.001
*R*-squared of the model 33%				

## Data Availability

The datasets used and/or analysed during the current study are available from the corresponding author upon reasonable request.
